# HIV Risk Perception and Constraints to Protective Behaviour among Young Slum Dwellers in Ibadan, Nigeria

**Published:** 2007-06

**Authors:** Adebola A. Adedimeji, Femi O. Omololu, Oluwole Odutolu

**Affiliations:** 1 Harvard School of Public Health, 665 Huntington Avenue, Building I, 12^th^ Floor, Boston, MA 02115, USA; 2 Department of Sociology, University of Ibadan, Ibadan, Nigeria; 3 Harvard AIDS Prevention Initiative in Nigeria, NAL House, Plot 990 NAL Boulevard, Area 3, Garki, Abuja, Nigeria

**Keywords:** HIV, HIV infections, Sexually transmitted diseases, Acquired Immunodeficiency Syndrome, Sexual behaviour, Risk perceptions, Slums, Epidemiology, Nigeria

## Abstract

This study examined the relationship between HIV/AIDS risk perception and protective behaviour among sexually-active urban young slum dwellers in Ibadan, Nigeria. The multistage sampling techniques were used for selecting 1,600 respondents aged 15-24 years. Of these, 1,042 (65%) respondents who reported unprotected sex in the last three months were selected for analysis. Although the sexually-active respondents demonstrated basic knowledge of HIV/AIDS and high risk perception, risky behaviour was common and protective behaviour was poor. About 48% of 505 males and 12% of 537 females had multiple partners. Similarly, 29% of males and 38% of females were engaged in transactional sex. Only 14% of males and 5% of females used any form of protection, resulting in the high rates of sexually transmitted infections reported by 27% of males and 10% of females. Structural and environmental constraints were identified as barriers to adopting protective behaviour. Therefore, programme and policy interventions should be designed to address the peculiar circumstances of urban young slum dwellers to curtail the HIV epidemic.

## INTRODUCTION

The rate of HIV prevalence in Nigeria is estimated to reach 18-26% by 2010 (1). This means that if the current trend is not checked, Nigeria will have the largest absolute number of people with HIV/AIDS in Africa within the next few years. Although available statistics do not provide accurate estimates of group prevalence, there is ample evidence to suggest that rates of infection are higher in urban areas and among young people aged 15-24 years (2,3). Thus, urban young people constitute a significant risk group.

The last 30 years witnessed an enormous increase in urban population in developing countries (4). Among other things, poor economic conditions make it difficult to adequately manage this increasing number of people, thus leading to economic and social inequality (5,6) and acute health problems because of an over-stretched healthcare infrastructure (7). Young people constitute a huge proportion of the urban population (4), and the need to focus on their sexual behaviour is informed by deteriorating living conditions, pervasive poverty (4,8), and the urban character of the HIV/AIDS epidemic (9). Zulu et al. (7), Ulin (10), and Carael and Allen (11) have provided evidence, suggesting that deteriorating economic and living conditions in urban areas have increased the likelihood that women, especially adolescent girls, will engage in behaviour which will make them susceptible to HIV infection/AIDS. Similar research has shown that low socioeconomic status and gender inequality explain the involvement of women in risky sexual behaviour, such as commercial sex work (12–14). Therefore, extreme conditions of poverty in urban slum communities may compel residents, especially adolescents, to engage in risky sexual behaviours. Despite the established link between economic deprivation and risk behaviours, no systematic investigation has yet been conducted to know how conditions of deprivation in urban slums influence HIV risk perception and sexual behaviour among adolescents in Nigeria.

Knowledge of AIDS and associated risks is almost universal among Nigerian adolescents, but how such knowledge influences risk perception and sexual behaviour among slum dwellers is not known. The disparity between knowledge and behaviour may be responsible for the continuing spread of the epidemic in Africa (15–21). It is, therefore, useful to examine how knowledge of AIDS influences people's construction of generalized and personalized risks (22) as this may increase our understanding of the disparity between knowledge and behaviour. Smith argued that “conception of risk in ethical and moral terms and the complex intertwining of collectively-shared moralities with individual assessments of ethics of personal behavior account for much of the seeming disconnect between what young people know about HIV/AIDS and what they do” (23 p.345).

Using qualitative and quantitative data, this study examined the relationship between knowledge of HIV/AIDS and risk perception and how this influences protective behaviour among sexually-active slum dwellers aged 15-24 years in Ibadan, Nigeria. The goal is to highlight the factors that influence risk perception and risk behaviour and also highlight barriers to adoption of protective behaviour.

## MATERIALS AND METHODS

### Study setting

Ibadan, one of the largest indigenous metropolitan areas in sub-Saharan Africa, has an estimated population of about two million coming from different parts of Nigeria and other parts of the world. The city, located on a major transport route to the northern parts of Nigeria, is the largest contemporary traditional Yoruba town.

The residential structure of the city can be divided into three homogenous groups: the core, the periphery, and the intermediate areas. The core area is the traditional area of the city, characterized by high levels of poverty, high density of population, lack of physical planning, dilapidated buildings, poor sanitation, inadequate health facilities, slum settlements, high level of illiteracy, and low level of socioeconomic activities. The intermediate areas, including Molete, Oke-Ado, Mokola, Eleyele, Agbowo, etc., are areas of late development, mainly inhabited by migrants from other Yoruba towns and ethnic groups, or those who moved out of family compound houses located in the tradtional areas of the city. The density of population here is lower than those of the traditional areas, and housing is also moderately scattered, although these are not well laid-out as those found in the peripheral areas. The periphery, including Old and New Bodija, University of Ibadan, Jericho, Iyaganku Government Reservation Areas, and other emerging well-planned areas of the city are inhabited mostly by the elite. These feature well laid-out residential apartments, low density of population, and essential social services. Healthcare needs of the population of the metropolis are served by the University College Hospital, two State hospitals, and several private medical facilities, in addition to traditional medical practitioners scattered all over the city.

Ibadan metropolis used to be under one local government—the Ibadan Municipal Government—before it was split into five local government areas (LGAs)—Southeast, Northeast, Southwest, Northwest, and North Central—in 1991. Respondents for the study were drawn from two of the five LGAs—Northeast and Southeast. These two LGAs contain the largest slum areas in the city. The characteristics of these two LGAs, which fit the criteria for slums, include high density of population, inadequate health, education and social facilities, poor sanitation, inaccessible road network, lack of potable water, and erratic electricity supply. Housing patterns show no distinction between buildings, located in large family compounds (with up to three or four families in one building). Similarly, leisure or recreational facilities are non-existent. The lack of recreational facilities is, perhaps, responsible for strong community organizations, such as cooperative societies that abound in the area and which enable residents to come together to implement community development activities.

The population structure consists of predominantly young people, with the majority aged 15-30 years, who work mostly as cobblers, seamstresses, tailors, and barbers and in other handicrafts. Although the population is predominantly Muslim, there is an active worship of deities. Overall, the majority of young people have some formal education, but many are currently out of school. Only a few communities have government health facilities and these are sparsely equipped. The only reliable health facility that residents patronize is the state-owned general hospital (Adeoyo Maternity Hospital is the nearest health facility owned by State government that is available to the communities. It is nearer than the more popular University College Hospital owned by Federal government, where special services are rendered), which is several kilometres away from many communities. Consequently, patent medicine stores (chemists) and itinerant medicine sellers serve the health needs of residents.

### Subjects

One thousand six hundred respondents were selected through the multistage sampling techniques. First, there was a purposive selection of the two LGAs—Northeast and Southeast—which contain the slum communities. Second, a mapping exercise was undertaken to generate a list of communities in the two LGAs. This mapping yielded a list of 72 communities from which eight were selected by systematic sampling. Third, a systematic sampling technique was also applied to select five enumeration areas from each of the eight communities based on a list of enumeration areas obtained from the National Population Commission. With this procedure, 40 enumeration areas were selected. Finally, 40 respondents equally divided between males and females, and age-groups of 15-19 and 20-24 years were selected from each enumeration area, making a total of 200 respondents from each community. The simple random techniques were used for selecting individual participants from a list of households containing at least one eligible respondent.

### Quantitative data

A self-administered questionnaire, containing 114 items on sexual experience, knowledge of reproductive health, knowledge of STIs/HIV/AIDS, knowledge of attitude trends and use of condom, risk perception, and health-seeking behaviour, was used for obtaining information. Respondents were briefed on the objectives of the study, and informed consents were obtained from them before interviews were commenced. All the respondents completed the interviews, and the interview was conducted in the local language. The average completion time was 50 minutes.

In total, 1,042 respondents (505 males and 537 females) representing 65% of the overall sample were selected as the working sample for analysis. The only inclusion criterion was reporting unprotected sexual activity in three months before the survey. Data from the questionnaire were processed using the EpiInfo software (version 6) and were analyzed using the SPSS software (version 12). Bivariate analysis assessed the differences that existed between males and females with respect to awareness of sexually transmitted infections (STIs)/HIV/AIDS, risk perceptions, risky sexual behaviour, and protective behaviour and identifying how males and females differ with respect to risk perception when they have unprotected sex with a casual partner or with a regular partner. Multivariate analysis, using logistic regression models, examined the relationship among background, knowledge, risk perception, and protective behaviour of respondents. The dependent variable—risky behaviour—was derived from a score of reporting any two or more of the following: unprotected sex with multiple partners, the last partner being non-regular, not using condom during the last intercourse, engaging in transactional sex, or having sex despite symptoms of STIs within three months before the survey. Respondents were coded ‘1’ if they reported any two or more of these and ‘0’ if otherwise. The logistic regression model was used for interpreting odds ratios. Odds greater than 1 indicated a greater likelihood of involvement in risky behaviour than for the reference category.

### Qualitative data

Focus-group discussions (FGDs), along with other participatory learning and action (PLA) activities, were conducted with male and female respondents in each community. Selection of participants for FGDs was based on identified homogeneous criteria, including residence within the community, age, and sex. Participants were selected from households to encompass both in-school and out-of-school respondents.

Overall, 16 FGD and PLA sessions were held (2 in each community with male and female participants). Two research assistants, along with the investigators, facilitated each session consisting of eight participants. Discussions were taped after consent was granted by participants. All discussions were conducted in the Yoruba language. Tape-recordings were transcribed and translated verbatim, and transcripts were reviewed for accuracy by re-listening to them while checking for anomalies. The ‘Open Code’ software was used for analyzing qualitative data. The coding focused on identifying consistent themes during the discussions, views from different transcripts were contrasted, and commonly-held perceptions were established. A validity check on this process was conducted, which involved the transcript being read by three persons who did not participate in the interviews, and there was complete agreement on the themes, which emerged. Findings from the qualitative data were used for complementing those from the quantitative data.

## RESULTS

Table 1 presents demographic and socioeconomic information about the respondents. Young adults, aged 20-24 years, made up the majority of the working sample. The large proportion of Muslim respondents reflected the dominant religion among residents in the study areas. Results on the educational status showed that about 2% of females had no formal education, whereas all males attained some level of formal education. Sixty-six percent of females as opposed to 49% of males were not in school at the time of the study. The majority of the respondents reported secondary education as the highest education attained at the time of the study. Due to poor economic conditions, young people were often engaged in income-generating activities either to meet their own needs or supplement family income. More females (53%) than males (47%) were involved in an income-generating activity. The majority lived with one or both parents. More females (41%) lived with relatives or their spouse (32%), while more males (19%) lived alone.

**Table 1:** Characteristics of respondents

Characteristics	All respondents (n=1,600)	Sexually-active respondents (n=1,042)	
		Male (n=505)	Female (n=537)
Age (years)			
15-19	50.0	37.2	35.9
20-24	50.0	62.8	64.1
Religion			
Muslim	57.4	63.7	59.0
Christian	41.5	35.3	39.1
Others	1.1	1.0	1.9
Marital status			
Single	83.7	11.3	37.1
Married	16.3	88.7	62.9
Currently in school			
Yes	54.6	50.9	33.4
No	45.4	49.1	66.6
Education			
None	0.6	-	1.7
Primary	14.3	10.0	23.0
Secondary	76.0	78.2	67.2
Others	9.1	11.8	8.1
Generation of income			
Yes	40.5	46.5	53.1
No	59.5	53.5	46.9
Living arrangement			
Parent(s)	72.8	72.6	57.0
Relative	8.5	7.8	9.8
Spouse	10.8	1.0	32.1
Alone	7.1	18.6	1.2

The results in Table 2 show almost universal awareness of STIs and HIV/AIDS among the respondents and also suggest a high prevalence of STIs among young slum dwellers. Although the finding—which should be interpreted with caution since it is not based on any clinical reports—that 27% of males and 10% of females reported being previously infected and 49% of males reported knowing another adolescent with an STI may indicate a high prevalence.

**Table 2:** HIV/AIDS knowledge, risk perception, and risky behaviour among respondents reporting recent unprotected sexual activity

HIV/AIDS awareness, risk perception, and practices	Males (n=505)	Females (n=537)
Awareness		
Heard of STIs	99.4	100
Heard of AIDS	97.6	99.6
Believe AIDS is real	99.0	99.1
Perception of risk		
Worried about getting infected with HIV/AIDS	57.8	36.3
Had STIs	27.3	9.1
Knew adolescent(s) infected with STIs	49.3	33.5
Knew someone infected with HIV/AIDS	17.2	9.3
Risk-taking sexual behaviour in last 3 months		
Had unprotected sex	100	100
Had sex with 2 or more partners	48.0	11.8
Received or given economic incentive for sex	29.4	38.4
Did not use condom during the last sex	93.4	90.3
Had sex despite symptom of STIs	9.7	15.8
Protective behaviour		
Initiated behaviour to avoid infection	14.7	4.5
Used condom during last intercourse	6.6	9.7

AIDS=Acquired immunodeficiency syndrome; HIV=Human immunodeficiency virus; STIs=Sexually transmitted infections

Table 2 shows that 58% of males and 36% of females were worried about being infected with AIDS, and this may be connected to the reported rate of STIs. Although risk perception was high, it did not appear to limit risk behaviours. For example, all the respondents selected for analysis recently had unprotected sex. Similarly, 48% of males and 12% of females reported unprotected sex with more than one partner during the three months before the survey. The disparity between risk perception and risky behaviours also manifested in the proportion of male (29%) and female (38%) respondents who reported engaging in an incentive-related sexual activity. Despite widespread awareness of STIs/HIV/AIDS and high level of risk perception, a few respondents (14.7% of males and 4.5% of females) initia-ted a protective behaviour to avoid infection.

**Fig. 1:**
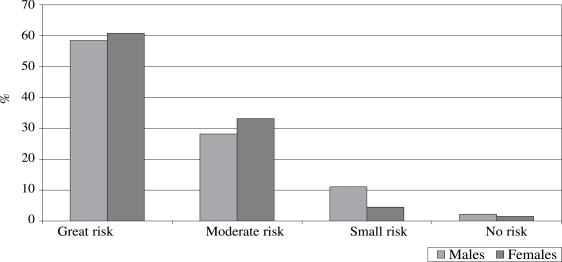
Perception of risk associated with unprotected sex with casual partners among sexually-active respondents

**Fig. 2:**
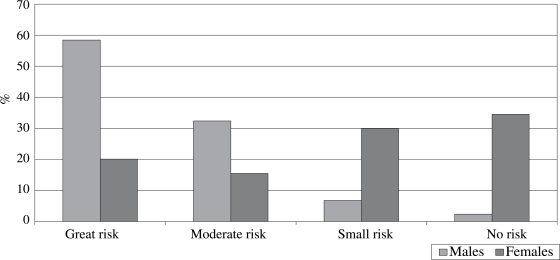
Perception of risk associated with unprotected sex with regular partners among sexually-active respondents

Perception of the respondents about the risks associated with unprotected sex varied by gender and type of partner. Nearly equal proportions of females and males reported a ‘great’ or ‘moderate’ risk in unprotected sex with casual partners (Fig. 1), but this perception differed when a regular partner was concerned (Fig. 2). In this case, nearly 60% of males, compared to 20% of females, reported a ‘great’ risk, while about 16% of females, compared to 30% of males, reported a ‘moderate’ risk. During the focus-group discussion sessions, females who mentioned ‘little’ or ‘no’ risk attributed this to having only one partner and trusting them, while those who mentioned ‘moderate’ or ‘high’ risk talked about lack of trust and inability to protect themselves. Males who reported ‘moderate’ or ‘high’ risk identified lack of trust as a reason.

As the level of risk perception was high and risky sexual behaviour was also common, a logistic regression analysis was undertaken to further examine the relationship between risk perception and risky behaviour. Those females who are married, those females who reported knowledge of mutual faithfulness, and those females living with a parental/authority figure (parents, relatives, or spouses) were significantly less likely to engage in risky sexual behaviour (Table 3). This, perhaps, reflects the social and cultural expectations of sexual behaviour of females in traditional societies. Although age, religious affiliation, current schooling status, involvement in an income-generating activity, having a high risk perception, and initiating a protective behaviour (mainly abstinence and condom-use) also reduced the odds of risky behaviour, the relationship was not significant.

**Table 3:** Odds ratios (standard errors) of risky behaviour among sexually-active females

Covariate and category	Model 1	Model 2	Model 3	Model 4
Background factors				
Age (years)				
20-24 (R)	-	-	-	-
15-19	0.99 (0.299)	0.91 (0.306)	0.88 (0.311)	1.00 (0.443)
Religion				
Christian (R)	-	-	-	-
Muslim	1.15 (0.278)	1.15 (0.281)	1.12 (0.285)	1.52 (0.394)
Marital status				
Single (R)	-	-	-	-
Married	0.20 (0.489)∗∗∗	0.18 (0.501)∗∗∗	0.17 (0.505)∗∗∗	0.11 (0.660)∗∗∗
Currently schooling				
No (R)	-	-	-	-
Yes	0.72 (0.314)	0.69 (0.320)	0.70 (0.325)	0.50 (0.459)
Generation of income				
No (R)	-	-	-	-
Yes	0.81 (0.336)	0.78 (0.343)	0.86 (0.348)	0.49 (0.490)
Living arrangement				
Alone (R)	-	-	-	-
Parents	0.11 (1.00)∗	0.10 (1.01)∗	0.10 (1.02)∗	0.04 (1.13)∗
Relative/spouse	0.12 (1.02)∗	0.12 (1.02)∗	0.12 (1.03)∗	0.14 (1.13)
Knowledge of preventive methods				
Abstinence				
No (R)		-	-	-
Yes		1.37 (0.308)	1.38 (0.313)	0.88 (0.420)
Mutual faithfulness				
No (R)		-	-	-
Yes		0.66 (0.284)	0.71 (0.307)	0.33 (0.421)∗∗
Avoid infected blood/blood products				
No (R)		-	-	-
Yes		1.23 (0.334)	1.34 (0.345)	1.26 (0.476)
Always use condom for sex				
No (R)		-	-	-
Yes		0.63 (0.391)	0.66 (0.307)	1.11 (0.512)
Avoid commercial sex workers				
No (R)		-	-	-
Yes		1.88 (1.07)	0.54 (1.07)	0.00 (21.68)
Avoid casual sex				
No (R)		-	-	-
Yes		0.70 (0.314)	1.41 (0.317)	0.82 (0.443)
Risk perception				
Ever thought you could be infected with STI?				
No (R)			-	-
Yes			1.97 (0.427)	1.92 (0.550)
Know adolescents infected with STI?				
No (R)			-	-
Yes			0.71 (0.317)	0.54 (0.435)
Worried about getting AIDS				
No (R)			-	-
Yes			1.19 (0.312)	0.96 (0.432)
I believe AIDS is curable				
No (R)			-	-
Yes			0.96 (0.277)	0.62 (0.394)
Protective behaviour				
Initiated preventive behaviour (abstinence)				
No (R)				-
Yes				0.96 (0.402)
Used condom during the last intercourse				
No (R)				-
Yes				0.54 (0.500)
2-Log likelihood	375.2	367.9	363.1	199.5

Levels of significance: ∗p<0.05; ∗∗p<0.01; ∗∗∗p<0.001 AIDS=Acquired immunodeficiency syndrome; R=Reference category; STI=Sexually transmitted infections

Those males who were currently schooling were involved in an income-generating activity and were worried about getting infected with AIDS were significantly more likely to engage in risky behaviours (Table 4). In addition, knowledge of certain preventive measures (abstinence, avoiding commercial sex, avoiding infected blood) and high risk perception also increased the odds of risky behaviours, although this was not significant. Generally, this finding suggests that, while male respondents had a high risk perception, they were more likely to engage in risky behaviours. Similarly, believing that AIDS is curable could also increase the tendency for risk-taking behaviour. For instance, those who reported that AIDS is curable were about 1.5 times more likely to engage in risky sexual behaviour.

**Table 4:** Odds ratios (standard error) of risky behaviour among sexually-active males

Covariate and category	Model 1	Model 2	Model 3	Model 4
Background factors				
Age (years)				
20-24 (R)	-	-	-	-
15-19	0.97 (0.207)	0.98 (0.209)	0.96 (0.215)	0.79 (0.237)
Religion				
Christian (R)	-	-	-	-
Muslim	1.38 (0.197)	1.46 (0.2001)∗	1.40 (0.205)	1.47 (0.228)
Marital status				
Single (R)	-	-	-	-
Married	0.61 (0.327)	0.62 (0.334)	0.65 (0.338)	0.52 (0.387)
Currently schooling				
No (R)	-	-	-	-
Yes	1.65 (0.247)∗	1.60 (0.253)	1.67 (0.257)∗	1.58 (0.294)
Generation of income				
No (R)	-	-	-	-
Yes	1.79 (0.248)∗∗	1.81 (0.254)∗∗	1.78 (0.256)∗	1.88 (0.292)∗
Living arrangement				
Alone (R)	-	-	-	-
Parents	0.97 (0.280)	0.99 (0.289)	0.99 (0.295)	0.89 (0.336)
Relative/spouse	1.22 (0.404)	1.15 (0.417)	1.14 (0.426)	0.22 (0.484)
Knowledge of preventive methods				
Abstinence				
No (R)		-	-	-
Yes		0.65 (0.203)∗	0.64 (0.212)∗	0.77 (0.239)
Mutual faithfulness				
No (R)		-	-	-
Yes		.081 (0.271)	0.83 (0.277)	0.72 (0.313)
Avoid infected blood/blood products				
No (R)		-	-	-
Yes		1.22 (0.264)	1.24 (0.284)	1.51 (0.315)
Always use condom for sex				
No (R)		-	-	-
Yes		1.33 (0.297)	1.28 (0.305)	1.42 (0.352)
Avoid commercial sex workers				
No (R)		-	-	-
Yes		1.65 (0.287)	1.63 (0.294)	1.59 (0.316)
Avoid casual sex				
No (R)		-	-	-
Yes		0.89 (0.216)	0.96 (0.223)	0.96 (0.245)
Risk perception				
Ever thought you could be infected with STI?				
No (R)			-	-
Yes			1.08 (0.248)	1.20 (0.280)
Know adolescents infected with STI?				
No (R)			-	-
Yes			0.97 (0.215)	1.00 (0.238)
Worried about getting AIDS				
No (R)			-	-
Yes			0.67 (0.216)	0.62 (0.244)∗
I believe AIDS is curable				
No (R)			-	-
Yes			1.45 (0.267)	1.23 (0.297)
Protective behaviour				
Initiated preventive behaviour (abstinence)				
No (R)				-
Yes				0.68 (0.243)
Used condom during last intercourse				
No (R)				-
Yes				0.69 (0.254)
2-Log likelihood	625.4	615.1	594.7	484.1

Levels of significance: ∗p<0.05; ∗∗p<0.01; ∗∗∗p<0.001 AIDS=Acquired immunodeficiency syndrome; R=Reference category; STI=Sexually transmitted infections

As these findings show, there was a contrast between high risk perception and risky sexual behaviours. The findings of the focus-group discussion sessions shed some light on the constraints and/or fatalism associated with transforming knowledge and perception of risk into preventive behaviour. For example, the participants reported:

*“Boys visit sex workers to avoid the commitment of maintaining a girl friend. They know that there is a risk of catching disease but they choose to ignore this …”* [Male, 20-24].

*“I don't worry about condoms because we only do it [sex] when I know I cannot get pregnant [safe period]. So to ask for condom now means I suspect him and that will bring trouble. Moreover, I don't expect him to infect me if he is not clean.* [not infected with an STI]” [Girl, 20-24]

Moreover, maximizing sexual pleasure has been cited as a reason for poor protective behaviour among men. Males alluded to this when they said:

*“I enjoy it [sex] if I do it naturally … so if I pay to enjoy myself, using condoms will reduce my pleasure. So, I immediately urinate or clean myself after ejaculation to avoid any infection if I don't trust the person.”* [Boy, 20-24]

These responses reveal the widely-held misconceptions and, perhaps, prevailing practices among males. The perception that STIs could be prevented by washing the genitals soon after ejaculation in unprotected sex was probably responsible for the reported rate of STI among half of males. Another plausible reason for the disconnection among knowledge, risk perception, and behaviour was the casual nature of sexual activity, and because sex was not anticipated, and young people were unable to plan for and take appropriate prevention, although they want to avoid negative consequences. The absence of social and recreational facilities in the slum communities was further highlighted as responsible for the idleness that characterize young people with little else to do rather than having sex as a means of recreation. Some participants noted:

*“There are no recreational facilities around here, so we have little to do when we return home in the evenings or during holidays. Boys are restless, you know…we always want to do something, so we turn to girls and before you know it, things [sex] are happening …”* [Male, 15-19]

*“NEPA [electricity] is not constant, so you cannot watch television/video or listen to radio. Again, there's no water, so you have to go somewhere to fetch water and you can only do that at night when you return home from school or your vocational centre. Some boys and girls take advantage of this to do what they want to do … young girls are unduly exposed in this community”.* [Female, 15-19]

Personality factors, concerns about self-image, cultural norms and inability to negotiate safe sex as a result of gender relations, and power disparities also limit ability of females to transform their knowledge into protective behaviour even when they have high risk perception. For instance, some participants noted,

*“… Even when you are aware of the risks [pregnancy], I cannot request that he uses condom. What would he think of me? A prostitute? I don't want anyone to think I am wayward, so, I cannot be carrying condoms around all the time.”* [Girl, 15-19]

Despite widespread knowledge about HIV/AIDS and its modes of transmission, high risk perception, and awareness of and access to condoms, many young people still do not take precautions to protect themselves during sex.

## DISCUSSION

There was a high level of risky sexual behaviour despite equally high levels of knowledge about HIV/AIDS and of condoms as a preventive measure. The level of risk behaviour was observed against a background of high risk perception, suggesting that urban young slum dwellers are aware of the risks associated with unprotected sexual activity but are constrained in adopting protective behaviours. This may be responsible for high rate of STIs, with serious implications for the spread of HIV/AIDS (24). The rate of STIs may also be due to inability to seek effective treatment and inadequate access to or complete absence of youth-friendly services in these settings. Thus, the existing gaps and constraints to seeking effective treatment of STIs must be addressed.

These findings also highlight the importance of addressing the wider sexual health challenges that confront young people in slum settings. In Nigeria, it is recognized that abstinence and consistent condom-use protect against STIs/HIV/AIDS. Condoms are widely available and are free for young people through many non-governmental organization outlets or at minimal subsidized prices. Yet, there is evidence that urban young slum people do not use these, suggesting the existence of barriers to adopting safe sexual practices. Both males and females fail to use or suggest condoms to their sexual partners because they are shy, or because suggesting condoms implies lack of trust for the sexual partner, or because they are concerned about the self-image, they project to their partners, parents, or the society at large. Therefore, some constraints to protective behaviour that females encounter are imposed by the social environment through social norms and gender-socialization practices that encourage women to be docile and men to be domineering in sexual matters. To this extent, society imposes risk on ‘good girls’ and ‘macho men,’ and it is important to understand the dynamics of condom-use behaviour so that strategies that enable young people to overcome barriers to improved sexual health can be developed and implemented. Moreover, making the social environment more supportive, for example, encouraging adults to support young people who are interested in adopting protective behaviours, such as condom-use, may prove effective in this regard.

The economic condition in slum communities is another reason why young people are unable to enact and sustain appropriate preventive measures despite engaging in risky sexual practices. Studies have documented the association between socioeconomic conditions and risky sexual practices (7,8,10,11,14,25). This body of evidence shows that economic deprivation considerably limit the ability to negotiate or adopt protective behaviour, especially among young women whose sexual partners are often older, richer, and more powerful men with whom they are unable to negotiate safe sex for fear of losing the economic benefits from such relationships. Among males, the evidence of increasing involvement in transactional sex with older women also raises questions regarding their ability to avoid risky sexual practices; further research is needed to uncover these. The evidence that transactional sex occurs, however, lends credence to the importance of poverty as a risk factor for young urban slum dwellers. Anthropologists examining the global AIDS pandemic have highlighted the impact of poverty and inequality as fundamental structural determinants of who is at risk (12,26). In the slum communities, such structural inequities have implications for the continuing spread of the AIDS epidemic, and this must be taken into account when designing both short- and long-term interventions.

Similarly, patterns of social organization, gender-roles/relations, and cultural norms regarding sexuality considerably affect the dynamics of sexual behaviour. Slum communities are closely knit, and the pattern of social organization largely mirrors those found in traditional societies. This close-knit structure is reflected in housing patterns organized along family compounds (agbo-ile) that are expected to regulate social and sexual behaviours of members, particularly those of females. Within this context, negotiating condom-use to prevent HIV/AIDS may signify negative connotations about individual character and sexual morality, and these threaten relationships. The ability of young women to request or insist on condom-use is, therefore, subjected to these constraints. Consequently, the combined effects of gender inequality, patterns of social/structural organization, and poverty, thus, put many young people in positions where they are unable to negotiate the terms of sexual relationships, or avoid risky behaviour despite having knowledge of risk.

The contrast in risk perceptions between casual and regular partners is striking. Despite being aware that unprotected sex with any partner involves some degree of risk, most females in regular/stable relationships did not associate any significant risk with their partners, either because they were unwilling to acknowledge that their partners posed a risk or because they were not fully aware of the sexual practices of their partners. Females might underestimate the risk posed by their regular partners based on their expectations of mutual fidelity; actual risk may, however, be elevated among such women because of the sexual behaviour of their partners as some studies have shown that male infidelity/multiple partnerships is tolerated in most African societies (27). Half of males and one in 10 females reported unprotected sex with multiple partners, some of whom, as previous research showed (28), are considered regular partners. Moreover, it is possible that even where individuals have multiple partners, so long as these relationships do not occur within institutionalized prostitution, risk may be severely under-estimated. It may also be related to moralistic perceptions through which individuals associate risk unto imaginary ‘immoral’ others (23). Moreover, as studies (29,30) suggested, it might be based on levels of AIDS-related morbidity and mortality within the wider community, which is not usually evident because of stigma. What is important, however, is that the perception of risk, within a context of stigma/morality or the extent of AIDS-related mortality, will have serious implications for the design and implementation of prevention strategies.

Of critical importance in the battle to reduce the effect of HIV/AIDS among the general populace and, indeed, among young people is the need to scale up the voluntary counselling and testing (VCT) component of HIV-prevention activities in Nigeria. Although this study did not investigate issues around knowledge of HIV sero-status and VCT, the findings underscore the importance of focusing on this component, particularly in view of the high levels of risk perceptions and risky sexual behaviours. Many young people were worried about getting infected with AIDS, but they failed to take the necessary steps, including testing, to avoid infection. Several reasons may be responsible for this. First is the fatalistic attitudes which many young people exhibit towards the epidemic. Second, information and services relating to VCT are currently available in hospitals and antenatal clinics that are not accessible to young people. Therefore, encouraging young people to use VCT services would require services to be provided where young people can have access. Integrating VCT services into the existing family life-education programmes in secondary schools and through community settings could facilitate a scale-up of the programme.

The above shows that having a correct perception of risk is insufficient to guarantee the adoption of protective behaviour. To successfully contain the spread of the epidemic among the poor in sub-Saharan Africa depends on the extent to which programme and policy intervention address peculiar structural-environmental and socioeconomic circumstances compelling young people to engage in risky sexual behaviours. Understanding the link among risk perceptions, risk-taking in sex, and preventive behaviour among young slum inhabitants and, indeed, the general population requires a careful consideration of the dynamics of sexual relationships, the motives that propel individuals to engage in sexual relationship and the structural-environmental factors that facilitate or hinder the adoption of preventive behaviour. While extensive documentation on the relationship between economic conditions and sexual behaviour exists, the link among slum residence, risk perception, and sexual behaviour has not thoroughly been investigated in Nigeria. Heise and Elias argued that the three-pronged approach of partner reduction, condom promotion, and treatment of STDs to prevent AIDS are inadequate to protect young people, especially females who are incapacitated in negotiating the terms of sexual encounters (31). Consequently, more qualitative research is needed to explore the peculiar structural and environmental circumstances that characterize risk perception, sexual relations, and protective behaviour among young slum people in Nigeria. In addition, research is needed with all categories of young people at risk to document the social dynamics of risk and the reasons for risk-taking and to develop their capacities to negotiate risk reduction when they are confronted with difficult circumstances. Empathic adult gate-keepers and researchers who are willing to facilitate change must also be identified and involved in this process and in disseminating the lessons learnt to the whole community.
